# The equity impact of brief opportunistic interventions to promote weight loss in primary care: secondary analysis of the BWeL randomised trial

**DOI:** 10.1186/s12916-019-1284-y

**Published:** 2019-03-01

**Authors:** J. Graham, K. Tudor, S. A. Jebb, A. Lewis, S. Tearne, P. Adab, R. Begh, K. Jolly, A. Daley, A. Farley, D. Lycett, A. Nickless, P. Aveyard

**Affiliations:** 1 Nuffield Department of Primary Care Health Sciences, Radcliffe Observatory Quarter, University of Oxford, Oxford, OX2 6GG UK; 2 Population Health Sciences, Bristol Medical School, University of Bristol, Canynge Hall, 39 Whatley Road, Bristol, BS8 2PS UK; 3 Institute of Applied Health Research, University of Birmingham, Birmingham, B15 2TT UK; 4 School of Sport, Exercise, and Health Sciences, Loughborough University, Loughborough, LE11 3TU UK; 5 Faculty Research Centre for Advances in Behavioural Science, Coventry University, Coventry, CV1 5FB UK

## Abstract

**Background:**

Guidelines recommend that clinicians should make brief opportunistic behavioural interventions to patients who are obese to increase the uptake of effective weight loss programmes. The objective was to assess the effect of this policy on socioeconomic equity.

**Methods:**

One thousand eight hundred eighty-two consecutively attending patients with obesity and who were not seeking support for weight loss from their GP were enrolled in a trial. Towards the end of each consultation, GPs randomly assigned participants to one of two 30-s interventions. In the active intervention (support arm), the GP offered referral to a weight management group. In the control intervention (advice arm), the GP advised the patient that their health would benefit from weight loss. Agreement to attend a behavioural weight loss programme, attendance at the programme and weight loss at 12 months were analysed by socioeconomic status, measured by postcode using the Index of Multiple Deprivation (IMD).

**Results:**

Mean weight loss was 2.43 kg (sd 6.49) in the support group and 1.04 kg (sd 5.50) for the advice only group, but these effects were moderated by IMD (*p* = 0.039 for the interaction). In the support arm, weight loss was greater in higher socioeconomic groups. Participants from lower socioeconomic backgrounds were more likely to accept the offer and equally likely to attend a weight loss referral but attended fewer sessions. Adjusting for these sequentially reduced the gradient for the association of socioeconomic status with weight loss from + 0.035 to − 0.001 kg/IMD point. In the advice only arm, 10% took effective action to promote weight loss. The decision to seek support for weight loss outside of the trial did not differ by socioeconomic status, but weight loss among deprived participants who used external support was greater than among more affluent participants (*p* = 0.025).

**Conclusion:**

Participants’ responses to GPs’ brief opportunistic interventions to promote weight loss differed by socioeconomic status and trial arm. In the support arm, more deprived people lost less weight because they attended fewer sessions at the programme. In the advice arm, more deprived people who sought and paid for support for weight loss themselves lost more weight than more affluent people who sought support.

**Trial registration:**

This trial is registered with the ISRCTN registry, number ISRCTN26563137. Date of registration: January 3, 2013; date of first participant recruited: June 4, 2014

**Electronic supplementary material:**

The online version of this article (10.1186/s12916-019-1284-y) contains supplementary material, which is available to authorized users.

## Introduction

The history of economic development shows that, broadly speaking, the prevalence of obesity rises with national wealth very probably because, as populations shift from rural to urban areas, the variety and amount of food available increases and manual tasks are replaced by automation [1, 2]. Obesity emerges first in the most affluent parts of society, but when a large proportion of the population become obese, a new trend is evident in which the most deprived have the highest prevalence of obesity [3]. This gradient contributes to the observed inequities in economic productivity, health outcomes and life expectancy. This situation calls for a wide-ranging and comprehensive policy response, designed to bring proportionally greater benefits to the most deprived groups. Part of the response will require interventions to treat established obesity if we are to avoid an unsustainable toll of morbidity and mortality in the next 50 years [4]. However, there are concerns that individually focused interventions, particularly those that rely on high levels of individual voluntary effort and organisation, termed agency, may exacerbate inequalities [5, 6].

Randomised controlled trials (RCTs) have established that modest weight loss can prevent weight-related morbidity and mortality [7, 8]. Although implementation in routine practice has proved challenging, RCTs have established that widely available commercially provided weight loss programmes can achieve greater weight loss and health benefits than self-management approaches, are cost-effective and can be cost-saving [9, 10]. In the UK, for example, some local areas can provide free referrals to community weight loss groups, usually for 12 weeks, as part of healthcare provision. Despite this, those from more deprived areas are less likely to use these community group weight loss programmes [11], even where participation is offered at no cost as part of a trial [12].

It has been hypothesised that interventions such as these weight loss programmes which require a high level of agency to enact may widen social inequalities [5]. Agency refers to motivation, organisation and capacity including material resources to enact behavioural responses. Social inequalities in the uptake of weight loss programmes, even when offered by GPs at no cost to the participant, all rely on agency to obtain health benefits. Inequalities in the response to intervention may arise at multiple stages of the pathway, including doctors offering a referral, patients’ acceptance of the referral, attendance at the programme, continued engagement with the programme and ability to enact the advice of the programme and continued attendance at it, all of which may affect eventual weight loss. Hence, despite being an effective weight management strategy at a population level, these weight loss programmes may also serve to increase inequalities between social groups.

We published an RCT showing that when GPs opportunistically endorse, offer and facilitate referral of unselected patients who were obese to a commercial weight management programme, this is well-received and results in greater weight loss at 1 year than when GPs advised weight loss alone [13]. In this pre-planned but exploratory subgroup analysis [14], we examine whether the outcome differed by deprivation and, if so, where in the pathway this occurred in both the support arm, where GPs suggested a referral, and the advice arm, where they simply advised weight loss would be beneficial. Both arms arguably require agency on the part of the participant to engage with and enact the advice offered in order to lose weight and improve health.

## Methods

### Study design and participants

The protocol and the primary outcome have been published previously [13, 14]. In brief, this study was a parallel, two-arm, randomised trial of a brief intervention for obesity conducted in primary care. Researchers screened consecutively attending patients waiting to see 137 different GPs across the south of England. We sought to enrol anyone who had a BMI ≥ 25 kg/m^2^ if they were Asian or ≥ 30 kg/m^2^ from all other ethnic groups, and 83% of such people agreed. We excluded people already attending weight loss programmes or those attending their GP for the purposes of weight loss support.

Participants had their consultation with the GP as normal and towards the end were randomised to one of two opportunistic brief behavioural interventions. In the ‘support’ arm, GPs endorsed, offered and facilitated a referral to one of two community weight management services, which were offered free to participants for 12 weeks. These services were provided commercially by Slimming World and Rosemary Conley. In the ‘advice’ (control) arm, GPs advised participants to lose weight to benefit their health. The aim was for GPs to deliver both interventions within 30 s. The trial had approval from the NHS Research Ethics Service and is registered ISRCTN: 26563137.

### Independent variable

The independent variable was socioeconomic status, measured here by the Index of Multiple Deprivation (IMD) score. IMD score is calculated based on census data for each lower level super output area (LSOA) that contains the participant’s postcode. Each area has an average of 1500 residents. The deprivation score is based on income, employment, education, health, crime, housing and living environment of people within that area. Each area is given a score from 1 to 100. A higher IMD score indicates higher levels of socioeconomic deprivation.

### Outcomes

For this analysis, we used the primary outcome of the trial, weight change between baseline and 12 months, and incorporated self-reported weight if the measured weight was missing. We weighed 1419 (75%) participants at 12 months and had self-reported weight on an additional 141 (7%). Otherwise, we imputed data using the baseline observation carried forward (BOCF) method for people whose weight was completely missing at 12 months (*n* = 320, 17%).

Researchers recorded whether participants accepted a referral at the time of the initial consultation, and therefore, there were no missing data. We obtained data from Slimming World on attendance for the majority of those accepting referrals (94%). Data was collected through routine systems and used to measure whether participants attended and the number of sessions attended for all participants that attended at least once.

We collected data by telephone or in-person interview on whether participants took action to lose weight at 3 and 12 months. Effective action was defined as taking action where there is evidence from trials that using that approach will lead to greater weight loss than self-directed weight loss efforts. We classified effective actions as attending a weight loss programme, prescription of orlistat or alli (orlistat bought without prescription), or following a total or partial meal replacement weight loss programme [15, 16].

### Statistical analysis

#### Did weight loss differ by levels of deprivation?

In this and all subsequent analyses, we used generalised linear mixed effects models with either an identity or logistic link function depending on whether the outcome was linear or binary. The randomisation was stratified by GP, so this term was added as a random effect and the link function was either a logistic term for binary outcomes or identity function for continuous outcomes. In this first analysis, we included baseline weight, trial arm, IMD score as an untransformed linear term and IMD × trial arm. The outcome variable was weight at 12 months. Having found evidence of moderation, we proceeded to analyse each arm separately to understand the cause of moderation.

#### Analyses within the support arm

Within the support arm, we examined whether the proportion of people accepting a referral when offered one by the GP differed by IMD score. The denominator was everyone in that arm. Among those who accepted a referral, we examined whether the proportion that attended at least one session and the number of sessions attended were associated with IMD score. Finally, we examined the association of IMD score with weight loss by adjusting sequentially for these terms to see whether this abolished the association between weight loss and IMD score.

#### Analyses within the advice arm

In the advice arm, we examined whether there was a difference by IMD score in participants who subsequently decided to use an effective form of weight loss support, mainly attending a commercial weight loss programme at their own expense. We also examined weight loss by IMD score, split by whether or not participants took effective action.

All statistical analyses were conducted according to the pre-specified statistical analysis plan using SPSS version 22. As these were predefined exploratory analyses, we mainly calculated and present 95% confidence intervals but present *p* values for analyses to help with the interpretation.

## Results

### Descriptive data

Between June 2013 and December 2014, 8403 patients were screened and 1882 were enrolled in the trial. Nine hundred forty participants were assigned to the support intervention and 942 to the advice intervention.

Participants had a mean age of 56.0 years (standard deviation (sd) 16.1), 1076 were women (57%) and 96 (5%) were from minority ethnic groups. The mean baseline weight was 92.5 kg (sd 15.3) for women and 104.6 kg (sd 15.7) for men, with mean BMI being 34.9 kg/m^2^ (sd 4.8). Mean IMD score was 15.7 (sd 11.8) in the advice group and 16.4 (sd 12.6) in the support group, and it ranged from 1.3 to 81.8. There was no evidence that people who declined participation in the trial differed from those who accepted in terms of age, gender, ethnic group or BMI, but data on postcode were deemed identifiers and not available for those who declined to participate.

**Fig. 1:**
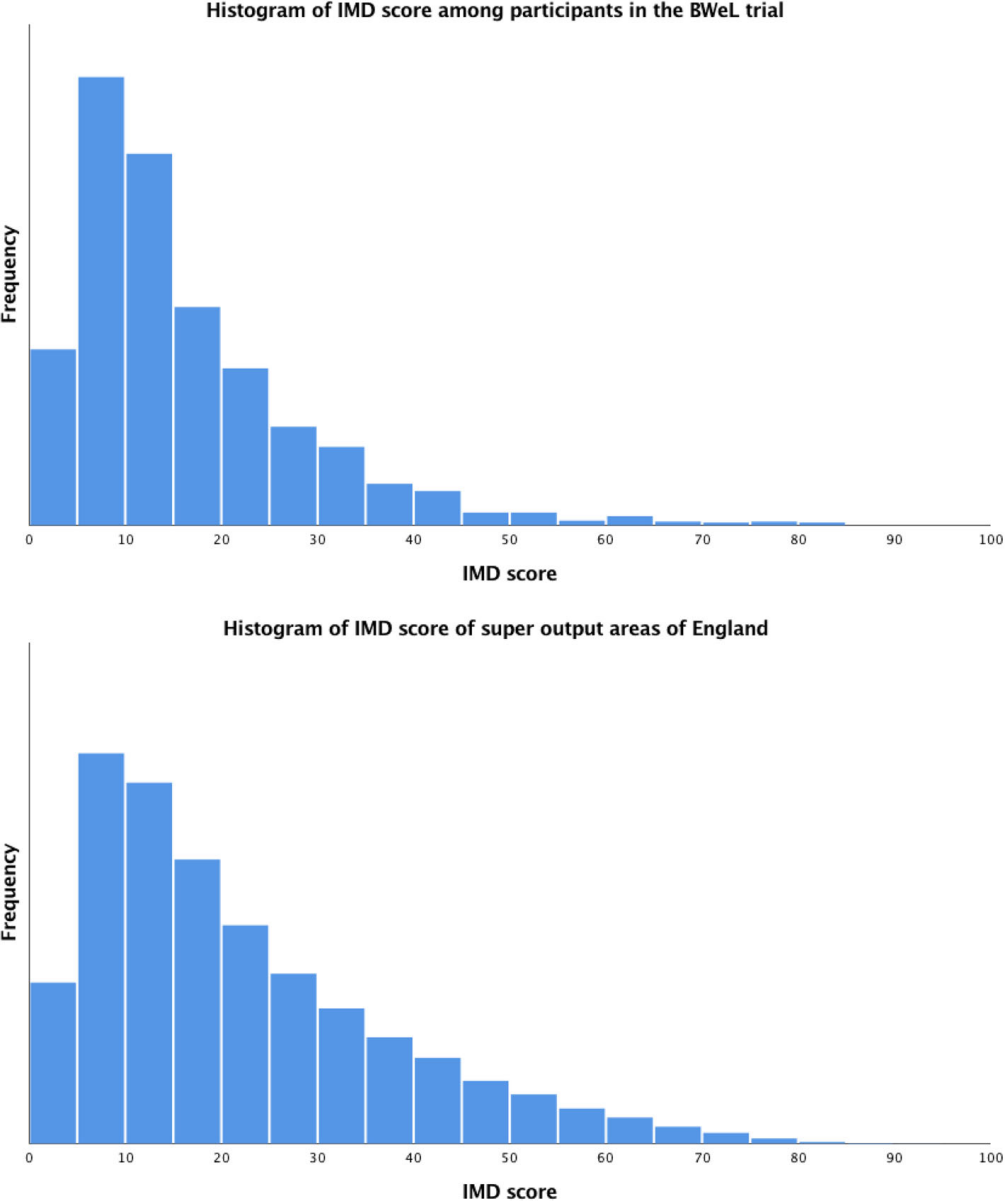
Frequency distribution of participants’ IMD score in the BWeL trial (top) and distribution of lower super output areas IMD score in England (bottom)*. *Higher scores represent greater deprivation

Figure 1 presents a histogram demonstrating the distribution of participants’ IMD score in the trial and the frequency of lower super output areas IMD scores shown by IMD decile (Office for National Statistics, 2015). The distribution of IMD scores was somewhat similar to that of England as a whole, but with a higher proportion of more affluent participants. IMD scores did not differ significantly between individuals who provided data at 12-month follow-up compared to those whose data was missing (*p* = 0.54).

Four hundred one participants in the support group (53% of those followed up) took effective action by 12 months, while 96 participants did so in the advice group (10%).

### Did level of deprivation moderate the effect of trial arm on weight loss?

**Fig. 2:**
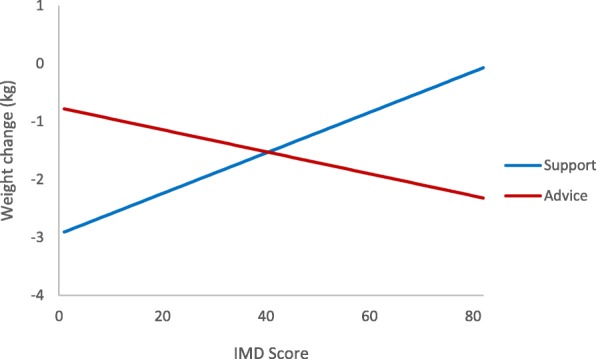
Weight change at 12 months in each trial arm by deprivation

At 12 months, weight loss was 2.43 kg (sd 6.49) in the support group and 1.04 kg (sd 5.50) in the advice group. IMD score was a significant moderator of the relationship between group and weight loss (IMD score × group coefficient was − 0.047, 95%CI − 0.09, − 0.02, *p* = 0.039). In the advice group, a higher proportion of deprived participants lost more weight at 12 months while in the support group, this relationship was reversed such that a higher proportion of deprived participants lost less weight (Fig. 2).

We therefore proceeded to analyse each arm separately to determine the possible causes of these differences. To check the models, we added square terms for deprivation but they did not improve the fit. We also plotted the mean weight loss in each decile of the IMD against the fitted regression line for the support and the advice arm, showing reasonable fit (see Additional file 1 Figure S1 and S2)

### The support arm

#### Did the acceptance of an offer differ by level of deprivation?

**Fig. 3:**
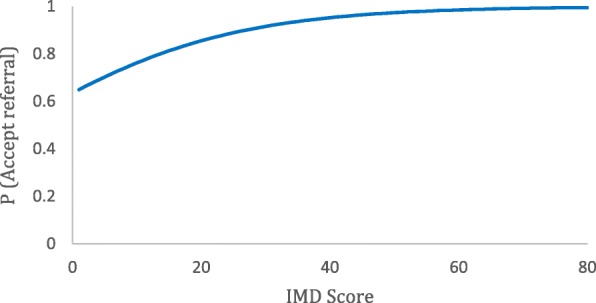
Proportion of participants accepting a referral to weight management

In the support group, 722 (77%) participants accepted a referral to weight management when offered by the GP. More deprived participants were more likely to accept the referral, odds ratio (OR) for a 10-point increase in IMD was 1.20 (95%CI 1.04 to 1.35, *p* = 0.015; Fig. 3).

#### Did attendance at a programme differ by level of deprivation?

**Fig. 4:**
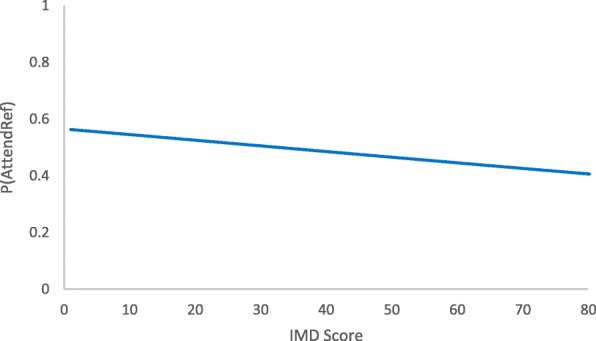
Proportion of participants who attended weight management having accepted a referral

Of those participants who accepted a referral to a commercial weight loss programme, 387 participants went on to attend the class (54%). Attendance following acceptance of referral was slightly lower among more deprived compared to less deprived participants (not statistically significant). The OR for attendance for a 10-point increase in IMD was 0.92 (95%CI 0.82 to 1.03, *p* = 0.17 Fig. 4). However, as more deprived patients were more likely to accept a referral, there was no evidence that attending at least one session differed by deprivation in the whole population, with an odds ratio of 1.00 (95%CI 0.90 to 1.12, *p* = 0.99) for a 10-point increase in IMD.

#### Did the number of subsequent attendances differ by level of deprivation?

**Fig. 5:**
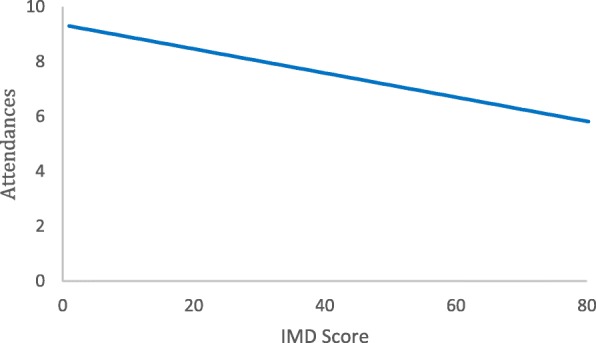
Mean number of sessions attended at the weight management service by participants in the support arm who attended at least one appointment

In those participants who attended at least one weight management session, the mean number of sessions attended was 8.0 (sd 3.7). People who were more deprived attended fewer sessions. For a 10-point increase in IMD score, the number of attendances declined by − 0.44 (95%CI − 0.8 to − 0.13, *p* = 0.006, Fig. 5).

#### Weight loss in those who declined the offer of weight loss support

Of those participants who were in the support group but did not accept the referral or attend a weight management at 12-month follow-up, there was no evidence that weight loss differed by levels of deprivation; weight loss was reduced by 0.18 kg (95%CI − 0.17 to 0.53, *p* = 0.30) for every 10-point increase in IMD.

#### Explaining the association between deprivation and weight loss in the support arm

The coefficient for the association between deprivation and weight change in the support arm was 0.035 (95%CI 0.002 to 0.068, *p* = 0.040). Adjusting for acceptance of referral slightly strengthened the association to 0.042 (95%CI 0.009 to 0.075, *p* = 0.012). Adding a term for whether or not participants attended at least one session reduced the coefficient slightly to 0.031 (95%CI − 0.008 to 0.069, *p* = 0.12). However, adjusting for the number of sessions attended reduced the coefficient to − 0.001 (95%CI − 0.061 to 0.062, *p* = 0.99).

### The advice arm

#### Taking effective action and effect on weight

In the advice group, 96 (10%) participants took effective action. There was no evidence this varied by levels of deprivation. The OR for a 10-point increase in IMD was 0.96 (95%CI 0.79 to 1.17, *p* = 0.71).

**Fig. 6:**
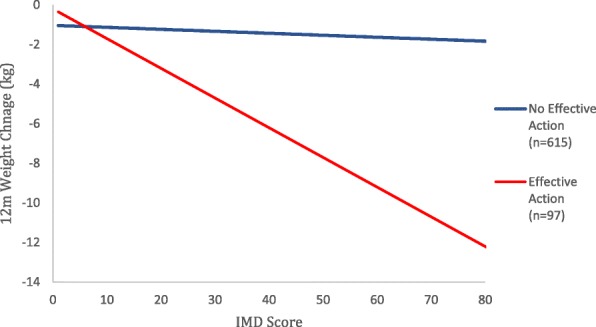
Weight change at 12 months for participants in the advice group taking effective action and not taking effective action by deprivation

We examined whether the association between weight loss and deprivation differed by whether or not participants took effective action by adding a multiplicative term for effective action × IMD score (Fig. 6). As this was significant (interaction coefficient = 0.14, 95%CI 0.018 to 0.27, *p* = 0.025), we examined the association between IMD and weight loss separately for those who did and did not take effective action in the advice arm. Among participants taking effective action, weight loss was somewhat but not significantly greater with increased deprivation; for every 10-point increase in IMD score, weight loss was 0.68 kg (95%CI 0.21 to − 0.072, *p* = 0.34) greater. Among those not taking effective action, the coefficient for the association between weight change and a 10-point increase in IMD was − 0.003 (95%CI − 0.043 to 0.036, *p* = 0.86) implying almost no association.

#### Explaining the association between deprivation and weight loss in the advice arm

The coefficient for the association between weight change and IMD was − 0.012 (95%CI − 0.042 to 0.019, *p* = 0.45). The strength of association was largely unchanged after adjusting for the use of an effective weight loss intervention (coefficient = − 0.016, 95%CI − 0.056 to 0.023, *p* = 0.42).

## Discussion

### Summary

Socioeconomic deprivation moderated the effect of a brief opportunistic behavioural intervention on weight change at 12-month follow-up. In the support group, less deprived participants lost more weight, while in the advice group, more deprived participants lost more weight. In the support group, socioeconomically deprived participants were more likely to accept the referral but attended fewer sessions than those who were less deprived. Adjusting for the number of attendances accounted for the relationship between deprivation and weight change in the support group. In the advice group, 10% of people took effective action to lose weight (predominantly via attending a commercial weight management programme). The probability of taking effective action did not differ by level of deprivation. However, weight loss among those taking effective action and who lived in deprived localities was much greater than among those taking action who lived in more affluent areas. Thus, more deprived participants were just as likely to take effective action when compared to less deprived participants but achieved greater weight loss by doing so.

### Strengths and limitations

The unique feature of this study is that the data come from a trial in which advice to lose weight or the offer of a referral to a weight management programme was given to the large majority of patients consulting a physician—a true test of opportunistic interventions delivered at scale. We might presume that most patients were not particularly motivated to lose weight, since we excluded patients who were already actively engaged in a programme or seeking help to lose weight from their doctor. It therefore provides the only data of its kind on the impact of opportunistic weight loss interventions on obesity-related inequalities, which current guidelines advocate. A strength of the study is the rate of follow-up data at 12 months (75% weighed, with an additional 8% reporting their weight), which is much higher than the typical follow-up rate of weight loss trials at 12 months (63%) [17].

A limitation of the study is that, for practical reasons, we mostly recruited general practices within 90 min’ drive of Oxford, UK. As such, most areas were more rural and more affluent than England as a whole. The only large conurbation that we recruited from was Bristol. There was no evidence of a difference in enrolment to the trial by age, gender or BMI, but we could not collect postcode, which we used to assess differences by deprivation score, on people who declined to participate. However, only 17% of potential participants declined to take part, meaning that any bias in the uptake by socioeconomic deprivation is unlikely to have greatly affected the associations we observed. Although we planned this exploratory analysis, we did not base the sample size calculation on the ability to detect associations by socioeconomic deprivation, in common with most trials. Moreover, we did not plan a complementary qualitative investigation to specifically understand the socioeconomic differences we observed. Finally, it should be noted that the IMD score used in the study represents the levels of deprivation based on participants’ reported postcodes. Thus, IMD indicates the deprivation of the geographical area in which the participants live, rather than the individuals themselves.

### Comparison with existing literature

A previous systematic review considered a range of interventions to promote healthy eating and reported on the effects on the outcome by socioeconomic status [18]. Among studies focused on ‘person’ interventions (i.e. individually based information and education), the results were mixed; eight studies suggested the intervention effect was lower in the more deprived, five found no evidence of a difference and five suggested better outcomes for the more deprived. While the interventions examined in this review differed from the brief interventions we studied, we too observed a mixed picture. In our study, advice to lose weight, but not providing support to do so, was associated with better outcomes in the more deprived populations, primarily because some deprived people paid for support to lose weight and were much more successful than more affluent people who did likewise. However, in the support arm, the GP actively offered their patients a free weight loss programme and booked the patient into the programme without the participant needing to do anything other than agree. This manifestly requires participants to exercise less agency than in the advice arm. Nevertheless, in this arm, people who were more deprived lost less weight than the more affluent. The results do not support the proposal that low agency interventions necessarily widen socioeconomic inequalities [5].

Cross-sectional research suggests that people from more deprived areas are less likely to use community weight management programmes [11]. Moreover, in a trial testing a commercial weight loss programme where people received a letter from their GP encouraging participation which comprised free treatment, people who lived in more deprived areas were less likely to enrol than their more advantaged peers [12]. In contrast, in the present analysis where the offer of referral was made in person by the GP, we found that patients living in more deprived areas were more likely to accept a referral. Offering the referral within a consultation led to a fourfold higher uptake. This suggests that a direct offer is not only more acceptable overall, but is particularly so to people in more deprived circumstances. In contrast to our findings in the support arm and the advice arm, another investigation of people referred by GPs to a commercial weight loss programme showed no evidence of socioeconomic differences in weight loss outcomes [12]. Taken together, it remains somewhat unclear whether commercial weight loss programmes have equal retention, and weight loss by social group and large-scale evaluations have not reported on this [19, 20].

### Implications for research and practice

Although this was a trial-based analysis, the aim was to assess the impact of current health policy in several countries, which advocates clinicians give opportunistic brief interventions to refer people to weight loss programmes. Given the high rate of recruitment into this trial and that GPs received only light-touch training, the trial represents the enactment of current health policy, which is otherwise largely unadopted [7, 8]. This is important because obesity is more prevalent in people living in more deprived circumstances, and mass provision of weight loss support is likely to be an important part of an effective public health response to the problem of obesity. Even modest weight loss reduces the incidence of weight-related morbidity and improves cardiovascular risk factors [21]. Ensuring people in more deprived areas are able to benefit from these services is a key component of a system of proportionate universalism to reduce inequalities [22]. Community weight management programmes are an effective intervention with the advantage of an established infrastructure to support mass delivery [23, 24]. However, if the provision of these services is to avoid widening inequalities, careful attention needs to be paid to the rollout of this support.

This analysis shows clearly that more deprived populations will gain greater benefit from in-person offers of support and facilitated access to services, rather than by a letter and likely also, by inference, informal advertising of services, which require a proactive response. Moreover, since most of the inequity arises because of poorer retention in the programmes, attention needs to be paid to the barriers to attendance and greater efforts by the programme providers themselves for their more deprived users. Previous research has explored potential barriers to initial attendance and adherence to community weight management programmes in populations from a range of socioeconomic backgrounds [25–27]. Frequently cited barriers have included cost [28, 29], work commitments [26] and childcare commitments [27]. However, there is a paucity of research into barriers that are specific to individuals from deprived areas. Thus, future research should aim to unpick specific facilitators and barriers in this population.

## Conclusion

When GPs actively offer brief opportunistic interventions to unselected patients who are obese, more deprived people seem more likely to accept support but attend less frequently and lose less weight than more affluent patients. However, when GPs offer advice to lose weight, subsequent use of support does not differ by levels of socioeconomic deprivation but weight loss is greater among the more deprived population.

## Additional file

Additional file 1: Evidence of linearity in the support arm and the advice arm. **Figure S1.** Weight loss at 12 months in support arm by socioeconomic status showing the fit of the line to the data. Figure S2. Weight loss at 12 months in advice arm by socioeconomic status showing the fit of the line to the data. (DOCX 60 kb)

## References

[1] Sallis JF (2016). Physical activity in relation to urban environments in 14 cities worldwide: a cross-sectional study. Lancet.

[2] Carrillo-Larco RM (2016). Obesity risk in rural, urban and rural-to-urban migrants: prospective results of the PERU MIGRANT study. Int J Obes.

[3] Monteiro C, Moura E, Conde W, Popkin B (2004). Socioeconomic status and obesity in adult population of developing countries: a review. Bull World Health Organ.

[4] Swinburn B, Dietz W, Kleinert S (2015). A Lancet Commission on obesity. Lancet.

[5] White, M., Adams, J. & Heywood, P. How and why do interventions that increase health overall widen inequalities within populations? in Social Inequalities and Public Health (ed. Babones, S.) 65–81 (Policy Press Scholarship Online, 2009).

[6] Tugwell P (2006). Health research profile to assess the capacity of low and middle income countries for equity-oriented research. BMC Public Health.

[7] Ma C, et al. Effects of weight loss interventions for adults who are obese on mortality, cardiovascular disease, and cancer: systematic review and meta-analysis. BMJ. 2017:j4849. 10.1136/bmj.j4849. 10.1136/bmj.j4849 PMC5682593 29138133

[8] Dunkley AJ (2014). Diabetes prevention in the real world: effectiveness of pragmatic lifestyle interventions for the prevention of type 2 diabetes and of the impact of adherence to guideline recommendations - a systematic review and meta-analysis. Diabetes Care.

[9] Ahern AL (2017). Extended and standard duration weight-loss programme referrals for adults in primary care (WRAP): a randomised controlled trial. Lancet.

[10] Parretti HM (2016). Clinical effectiveness of very-low-energy diets in the management of weight loss: a systematic review and meta-analysis of randomized controlled trials. Obes Rev.

[11] Relton C (2013). Deprivation, clubs and drugs: results of a UK regional population based cross sectional study of weight management strategies.

[12] Ahern AL, Aveyard P, Boyland EJ, Halford JCG, Jebb SA (2016). Inequalities in the uptake of weight management interventions in a pragmatic trial: an observational study in primary care. Br J Gen Pract.

[13] Aveyard P (2016). Screening and brief intervention for obesity in primary care: a parallel, two-arm, randomised trial. Lancet.

[14] Lewis A (2013). A brief intervention for weight management in primary care: study protocol for a randomized controlled trial. Trials.

[15] Astbury NM (2018). Doctor Referral of Overweight People to Low Energy total diet replacement Treatment (DROPLET): pragmatic randomised controlled trial. BMJ.

[16] LeBlanc AG (2012). Systematic review of sedentary behaviour and health indicators in the early years (aged 0–4 years). Appl Physiol Nutr Metab.

[17] Elobeid MA (2009). Missing data in randomized clinical trials for weight loss: scope of the problem, state of the field, and performance of statistical methods. PLoS One.

[18] McGill R (2015). Are interventions to promote healthy eating equally effective for all? Systematic review of socioeconomic inequalities in impact. BMC Public Health.

[19] Ahern AL, Olson AD, Aston LM, Jebb SA (2011). Weight watchers on prescription: an observational study of weight change among adults referred to weight watchers by the NHS. BMC Public Health.

[20] Stubbs RJ, Morris L, Pallister C, Horgan G, Lavin JH (2015). Weight outcomes audit in 1.3 million adults during their first 3 months’ attendance in a commercial weight management programme. BMC Public Health.

[21] Zomer E (2016). Interventions that cause weight loss and the impact on cardiovascular risk factors: a systematic review and meta-analysis. Obes Rev.

[22] Marmot M, Bell R (2012). Fair society, healthy lives. Public Health.

[23] Hartmann-Boyce J, Johns DJ, Jebb SA, Summerbell C, Aveyard P (2014). Behavioural weight management programmes for adults assessed by trials conducted in everyday contexts: systematic review and meta-analysis. Obes Rev.

[24] Jebb SA (2011). Primary care referral to a commercial provider for weight loss treatment versus standard care: a randomised controlled trial. Lancet.

[25] Johns, D.J et al. Managing overweight and obese adults: evidence review. (2013).

[26] Gray CM (2013). Football Fans in Training: the development and optimization of an intervention delivered through professional sports clubs to help men lose weight, become more active and adopt healthier eating habits. BMC Public Health.

[27] Lavin JH (2006). Feasibility and benefits of implementing a slimming on referral service in primary care using a commercial weight management partner. Public Health.

[28] Ahern AL, Boyland EJ, Jebb SA, Cohn SR (2013). Participants’ explanatory model of being overweight and their experiences of 2 weight loss interventions. Ann Fam Med.

[29] Thompson R, Thomas D (2000). A cross-sectional survey of the opinions on weight loss treatments of adult obese patients attending a dietetic clinic. Int J Obes Relat Metab Disord.

