# Supplementary material for: The equity impact of brief opportunistic interventions to promote weight loss in primary care: secondary analysis of the BWeL randomised trial
Source: BMC Med. 2019 Mar 1;17:51. doi: 10.1186/s12916-019-1284-y (PMC6396456; doi:10.1186/s12916-019-1284-y)
Supplement: Supplementary file 1 — Evidence of linearity in the support arm and the advice arm. Figure S1. Weight loss at 12 months in support arm by socioeconomic status showing the fit of the line to the data. Figure S2. Weight loss at 12 months in advice arm by socioeconomic status showing the fit of the line to the data. (DOCX 60 kb) [file 12916_2019_1284_MOESM1_ESM.docx]

**Additional File 1 Evidence of linearity**

**Support arm**

We examined whether there was evidence that the association between IMD score and weight loss was non-linear by including a square term. As it did not add significantly to the model, (IMDscore*IMDscore coefficient was 0.001, p=0.098), we omitted it for parsimony. We also checked the fit of the model to the data by calculating the median weight loss for each decile of the IMD distribution (Figure 2).

**Additional File 1 Figure S1 Weight loss at 12 months in Support Arm by socioeconomic status showing the fit of the line to the data**

**Advice arm**

We examined whether there was evidence that the association between weight loss and IMD score was not linear by adding a square term but as this did not add significantly to the model fit (coefficient= -0.001, p=0.069), we proceeded with a linear term for IMD. We also checked the fit of the model to the data by calculating the median weight loss for each decile of the IMD distribution (Figure 6).

**Additional File 1 Figure S2 Weight loss at 12 months in Advice Arm by socioeconomic status showing the fit of the line to the data**
